# Unprogrammed Deworming in the Kibera Slum, Nairobi: Implications for Control of Soil-Transmitted Helminthiases

**DOI:** 10.1371/journal.pntd.0003590

**Published:** 2015-03-12

**Authors:** Julie R. Harris, Caitlin M. Worrell, Stephanie M. Davis, Kennedy Odero, Ondari D. Mogeni, Michael S. Deming, Aden Mohammed, Joel M. Montgomery, Sammy M. Njenga, LeAnne M. Fox, David G. Addiss

**Affiliations:** 1 Parasitic Diseases Branch, Centers for Disease Control and Prevention, Atlanta, Georgia, United States of America; 2 Division of Global HIV/AIDS, Centers for Disease Control and Prevention, Atlanta, Georgia, United States of America; 3 Kenya Medical Research Institute, Centre for Global Health Research, Nairobi, Kenya; 4 Public Health Department, School Health Program, Nairobi County, Nairobi, Kenya; 5 Division of Global Health Protection, Centers for Disease Control and Prevention, Nairobi, Kenya; 6 Eastern and Southern Africa Centre of International Parasite Control, Kenya Medical Research Institute, Nairobi, Kenya; 7 Children Without Worms, Task Force for Global Health, Decatur, Georgia, United States of America; Rosetta Genomics, ISRAEL

## Abstract

**Background:**

Programs for control of soil-transmitted helminth (STH) infections are increasingly evaluating national mass drug administration (MDA) interventions. However, “unprogrammed deworming” (receipt of deworming drugs outside of nationally-run STH control programs) occurs frequently. Failure to account for these activities may compromise evaluations of MDA effectiveness.

**Methods:**

We used a cross-sectional study design to evaluate STH infection and unprogrammed deworming among infants (aged 6–11 months), preschool-aged children (PSAC, aged 1–4 years), and school-aged children (SAC, aged 5–14 years) in Kibera, Kenya, an informal settlement not currently receiving nationally-run MDA for STH. STH infection was assessed by triplicate Kato-Katz. We asked heads of households with randomly-selected children about past-year receipt and source(s) of deworming drugs. Local non-governmental organizations (NGOs) and school staff participating in school-based deworming were interviewed to collect information on drug coverage.

**Results:**

Of 679 children (18 infants, 184 PSAC, and 477 SAC) evaluated, 377 (55%) reported receiving at least one unprogrammed deworming treatment during the past year. PSAC primarily received treatments from chemists (48.3%) or healthcare centers (37.7%); SAC most commonly received treatments at school (55.0%). Four NGOs reported past-year deworming activities at 47 of >150 schools attended by children in our study area. Past-year deworming was negatively associated with any-STH infection (34.8% vs 45.4%, p = 0.005). SAC whose most recent deworming medication was sourced from a chemist were more often infected with *Trichuris* (38.0%) than those who received their most recent treatment from a health center (17.3%) or school (23.1%) (p = 0.05).

**Conclusion:**

Unprogrammed deworming was received by more than half of children in our study area, from multiple sources. Both individual-level treatment and unprogrammed preventive chemotherapy may serve an important public health function, particularly in the absence of programmed deworming; however, they may also lead to an overestimation of programmed MDA effectiveness. A standardized, validated tool is needed to assess unprogrammed deworming.

## Author Summary

In countries with endemic soil-transmitted helminth infections, deworming medications are widely available from multiple sources, including over the counter. However, in many countries, national programs already provide deworming medications in mass drug administrations to primary school students, as part of World Health Organization recommendations. Evaluations of the effectiveness of such medications at reducing worm burden in children is based solely on the national program’s distribution schedules, primarily because little is known about how frequently deworming medications are obtained from other sources. We investigated sources of deworming medications received by children in a Kenyan slum, finding that more than half of school-aged and preschool-aged children received deworming medications outside of a national school-based deworming program. These drugs were received from multiple sources, including chemists, healthcare centers, and at schools, via the efforts of non-governmental organizations. These data strongly indicate a need to collect data on all sources of deworming medications when evaluating the effectiveness of national school-based deworming programs.

## Introduction

Soil-transmitted helminth (STH) infections affect approximately 2 billion persons worldwide [1], with school-aged children generally having the highest-intensity infections and highest prevalence of infection [2–6]. Improper disposal of human feces contaminated with helminth eggs exposes humans to infection following ingestion of eggs (*Trichuris trichura*, or whipworm, and *Ascaris lumbricoides*, or roundworm) or skin contact with larvae that hatch from eggs (*Ancystoloma duodenale* and *Necator americanus*, or hookworm). A wide array of physical effects have been attributed to intestinal STH infections, including anemia (primarily from hookworm infection) [7–9], Vitamin A deficiency [10], decreased physical fitness [11], decreased cognitive function [12, 13], decreased growth [12, 14–16], and intestinal obstruction [17]. Morbidity is directly related to infection intensity [18].

Without meaningful improvements in sanitation infrastructure in low-resource settings, elimination of STH infections is likely not feasible. Because of this, and because most morbidity is attributable to high- and moderate-intensity infections, the World Health Organization (WHO) recommends, rather than elimination, reduction of worm burden in individuals [19]. In 2001, WHO set the goal of providing regular preventive deworming chemotherapy to at least 75% of at-risk school-aged children by 2010, and urged endemic countries to develop programs to administer these drugs through schools and primary healthcare systems [20]. In 2012, pharmaceutical companies GlaxoSmithKline and Johnson & Johnson agreed to donate billions of doses of anthelminthic drugs to countries in need [21], enabling the expansion of existing government-sponsored deworming programs, most of which have not yet reached the 75% target.

Many STH-endemic countries, including Kenya, are now planning or actively implementing national school-based deworming programs, provided as mass drug administration (MDA) conducted by Ministries of Health, Ministries of Education, and nongovernmental organization (NGO) partners [22]. Based on STH prevalence mapping and spatial modeling in Kenya [23–25], a phased-in approach to school-based deworming was planned for selected districts in five of the country’s eight provinces, excluding Nairobi, Rift Valley, and North Eastern Provinces [22, 26]; this program is in the process of being implemented [26]. However, smaller-scale deworming programs are also frequently carried out by other in-country partners [27], who may use different regimens for deworming. In addition, deworming drugs are widely available from clinics, drugstores, and other sources. This ‘unprogrammed deworming’—deworming outside of the context of a nationally-administered STH control program—is frequently neither documented nor reported to health officials [27].

There is currently great interest in evaluating the effectiveness of national deworming MDA programs and progress towards the WHO treatment coverage targets for 2020 [28–31]. However, accurate reporting of deworming is required to monitor this progress. The unknown extent and patterns of unprogrammed deworming challenge both the monitoring and evaluation of STH control programs. We describe unprogrammed deworming in Kibera, an urban slum in Nairobi, Kenya. Nairobi is not included in the national deworming program, due to an overall low prevalence of STH infection [32].

## Methods

This study was conducted in two villages of Kibera, Kenya, during April—June 2012 as part of a larger cross-sectional evaluation of STH infection and nutritional status in children enrolled in the Centers for Disease Control and Prevention’s (CDC) International Emerging Infections Program (IEIP)/Kenya Medical Research Institute (KEMRI) surveillance platform. Details of this study are provided elsewhere [33]. In brief, IEIP enrolls all adults and children with head-of-household consent who have been living in all households in the two-village study area continuously for at least four months. Among households in the IEIP participant registry, approximately 25% were designated as potential sources for enrollment of preschool-aged children (PSAC) (aged 12–59 months) and infants (aged 6–11 months). The other 75% of households were designated as potential sources for enrollment of school-aged children (SAC) (aged 5–14 years); this ensured that two children were not selected from the same household. Households were selected with probability proportional to size from each target group, and one child was chosen randomly from each selected household. Up to three stools were collected from all selected children and tested by Kato-Katz analysis as described previously [33]. Field workers visited households and, using a visual recall aid showing the locally-available deworming medicines, asked parents about whether or not their SAC or PSAC had been dewormed in the past year, and the source of their deworming medication the last time they were treated in the past year. In addition, parents provided information on the school attended by the child. Schools were defined as ‘public’ (under the purview of the Ministry of Education), ‘private’ (privately-owned and operated, following public curricula), or ‘informal’ (not under the purview of the Ministry of Education and with independent curricula).

Because responses suggested that many children were receiving deworming medication at school, administrators and teachers were interviewed at five schools attended by children in the survey. These semi-structured interviews included questions about previous deworming and other health interventions that took place at the school (frequency of interventions, medications administered, and deworming partners). Information from these interviews was used to identify organizations providing deworming treatments in schools in this area. These organizations were subsequently contacted and asked to share any available deworming records from 2010–2012, including school name, school type and location, date of deworming event(s), enrollment figures, and treatment figures. Only 2012 data are included in this report. Drug coverage was estimated by dividing the number of doses of deworming medication reported to have been distributed at each deworming event by the total school enrollment at the time of deworming. Proportions were compared using chi-squared or Fisher’s exact test, where appropriate; missing data were excluded. Data were analyzed in SAS 9.3 (English).

### Ethics statement

This study was approved by Institutional Review Boards at the Kenya Medical Research Institute (KEMRI) and the U.S. Centers for Disease Control and Prevention (CDC). Written informed parental or guardian consent was required for all participants. Written assent was additionally obtained from all participants aged 13 years or older.

## Results

### Self-reported deworming

**Fig 1:**
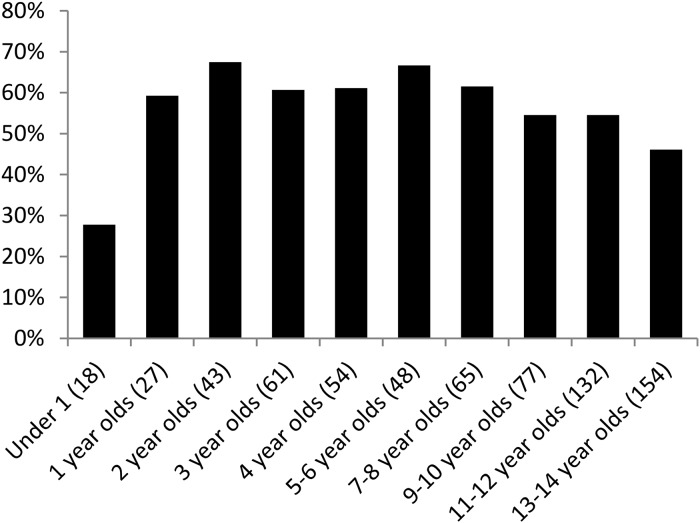
Proportion of children in each age group whose parents reported that they had been dewormed at least once during the previous year, Kibera, Kenya, 2012. Numbers in parentheses represent denominators for each age group.

Of the 692 children included in the initial study, 679 had data on past-year deworming, including 18 infants, 184 PSAC, and 477 SAC. Of the 679, 377 (55.5%) reported receiving deworming drug treatments during the previous year. Past-year deworming occurred approximately equally among PSAC (62.0%) and SAC (54.1%) (p = 0.07), a median of three months (range, 0–12 months) before the interview for both groups. Five (27.7%) infants received deworming medication during the previous year. We examined frequency of past-year deworming by one- and two-year age categories; when infants were excluded, there were no differences between these age groups (p = 0.12) (Fig. 1).

Among the 377 children dewormed during the previous year, the most common source of the most recent deworming medication was school (39.5%), a chemist (independently-owned commercial drug kiosks) (27.9%), or a clinic/hospital or health center (26.3%) (Table 1). Among PSAC who were dewormed, the chemist (48.3%) and clinic/hospital or health center (37.7%) were the most common source of deworming medications; most SAC who were dewormed (55.0%) received the drugs at school. The five infants who were dewormed received drugs from a clinic/hospital or health center.

**Table 1:** Sources of drugs for the most recent deworming, overall and by child type, among children who were reported as taking deworming medications at least once during the past year, Kibera, Kenya, 2012.

	Overall (n = 377)	Infants (n = 5)	PSAC (n = 114)	SAC (n = 258)
**School**	149 (39.5%)	0	7 (6.1%)	142 (55.0%)
**Chemist**	105 (27.9%)	0	55 (48.3%)	50 (19.4%)
**Clinic / hospital / health center**	99 (26.3%)	5 (27.8%)	43 (37.7%)	51 (19.8%)
**Medical camp (traveling health clinic)**	12 (3.2%)	0	4 (3.5%)	8 (3.1%)
**Individual mobile vendor**	7 (1.9%)	0	3 (2.6%)	4 (1.6%)
**Other**	5 (1.3%)	0	2 (1.8%)	3 (1.2%)

### School-based deworming of school-aged children in Kibera

Of the 477 SAC, 442 (92.7%) normally spent the day at school, while 85 (46.2%) of the 184 PSAC spent the day at a nursery school or early childhood learning center. At least 150 different schools were named by respondents as being attended by the SAC included in our study; school name was not recorded for eight SAC. Of the 71 schools for which data were available, 45 (63.4%) were informal, 16 (22.5%) were private, and 10 (14.1%) were public. Of the 393 school-aged children with data on schools attended, 194 (49.4%) attended a public school, 140 (35.6%) attended an informal school, and 59 (15.0%) attended a private school.

Among 383 SAC with data on both school type and deworming, those who attended a public school were more likely to have taken deworming drugs in the past year (from any source) (120/190, 63.2%) than children who attended a private school (29/58, 50.0%)(p = 0.07) or an informal school (61/135, 45.2%) (p = 0.001). Among all SAC who were dewormed, public-school children were non-statistically-significantly more likely to have received their most recent deworming medications at school (78/120, 65.0%) than private-school children (16/29, 55.2%) or children attending an informal school (30/61, 49.2%) (p = 0.11).

Of the 679 children in this analysis, 268 (39.5%) were infected with at least one STH, including *Trichuris* (n = 176; 25.9%), *Ascaris* (n = 156; 23.0%), and hookworm (n = 1; <1%). Past-year deworming was associated with reduced frequency of any STH infection (34.8% vs. 45.4%, p = 0.005). When data were limited to the three most common sources of deworming medications (school, chemist, and clinic/hospital/health center), SAC whose most recent deworming medication was sourced from a chemist were more frequently infected with *Trichuris* (19/50, 38.0%) than those whose most recent deworming medication was obtained from a clinic/hospital/health center (9/52, 17.3%) or school (33/143, 23.1%) (p = 0.05). The presence of other STH infections and any STH infection were not significantly different by treatment source among SAC or PSAC.

### NGO deworming of schools

Four NGOs were identified as providing school-based deworming medications to children in our study area and were contacted for follow-up. Local government in Nairobi County had partnered with one of these NGOs to deworm children at selected schools (independent from the Kenyan national school-based deworming program); the other three NGOs worked independently from the government and directly with schools. The NGOs reported deworming at 47 schools in Kibera during 2012; 44 (93.6%) schools received deworming treatment once and three were dewormed twice (a total of 50 deworming events). Of the schools with two deworming events during 2012, two had both events administered by the same NGO, and one received deworming treatments from two different NGOs, during February and December 2012. Calculated deworming coverage among enrolled pupils at these events ranged from 72%–130% (median 98.7%). Of 31 schools with data on school type, 19 were informal, eight were public, and four were private.

## Discussion

Unprogrammed deworming, defined as treatment with deworming drugs outside the context of a nationally-administered STH program, is increasingly recognized in many areas that are endemic for STH infections. Our data indicate that more than half of all preschool- and school-aged children in two villages of the informal settlement of Kibera, Kenya received unprogrammed deworming treatments during 2012. These treatments were obtained from a wide variety of sources, which differed by age group: while school-aged children most often obtained treatments in school, frequently through the efforts of NGOs, preschool-aged children more often received treatments from independent suppliers, such as clinics and chemist shops. The median time since last deworming was three months, suggesting that children may be treated several times each year. Because our survey questions were designed to identify only the most recent source of deworming medication, these data likely underestimate the true frequency of unprogrammed deworming events.

Although unprogrammed deworming is rarely reported, it is likely widespread. A recent evaluation of MDA in Bangladesh described high levels of unprogrammed deworming among school-aged children living in an area already receiving programmed school-based deworming [34]. The relatively low cost of such drugs also enhances their accessibility [34, 35]: at the local medical clinic in Kibera, albendazole and mebendazole cost approximately $0.02 USD for a single mebendazole tablet to approximately $0.22 USD for albendazole suspension (O. Mogeni, personal communication). Although individual-level deworming is indicated in specific circumstances, such as for children with palmar pallor in Integrated Management of Childhood Illness (IMCI) programs [36, 37] or in certain maternal health settings, mass unprogrammed deworming, when carried out in an area that already has programmed deworming, may represent wasted treatment in areas with already-limited resources.

In addition to the potential for wasted treatments, unprogrammed deworming may complicate evaluations of effectiveness of nationally-implemented STH control programs. Nonreporting of deworming events to Ministries of Health or to WHO, as previously reported, [27], compromises the monitoring of progress towards WHO-recommended anthelmintic coverage targets [18,20]. Typically, changes in prevalence and intensity of STH infection are assumed to be due to the effectiveness of drugs delivered through programmed MDAs [38–42], occasionally in combination with improvements in sanitation or hygiene [43]. However, high levels of unprogrammed treatment may inflate the apparent effectiveness of programmed MDA. In our study, 55% of children received unprogrammed deworming drugs, a proportion not far below the 75% coverage target set by the WHO [20]. Unprogrammed deworming at this level would almost certainly have an impact on the evaluated effectiveness of a nationally-run MDA program. While the mutual influences of unprogrammed deworming and programmed MDA on each other’s administration frequencies are unknown, in the Bangladesh study, unprogrammed deworming continued at a high rate despite the presence of a national school-based deworming program: 38.7% (95% CI 51.9–64.4) of school-aged children living in a district already receiving two programmed school-based deworming events during 2009 [during which surveyed coverage was 52.3% (95% CI 43.6–61.1%) and 54.3% (95% CI 44.8–63.8%)] reported that they had additionally obtained deworming drugs during that year from other, non-school sources [34]. The lack of coordinated timing of these unprogrammed treatments, even where >1 treatment per year is appropriate (for example, in very high STH-prevalence settings) [18], could also influence their effectiveness.

The effectiveness of unprogrammed deworming on the control and transmission of STH infections is unclear. Nairobi is not considered a high-prevalence region for STH infections [32], and therefore Kibera is not included in the Kenyan national school-based deworming program. Receipt of unprogrammed deworming was associated with reduced STH infection in our study area, and heavy infections were very rare [33]; this may be due in part to the unprogrammed deworming events reported by participants. However, unprogrammed deworming may expose infected individuals to drugs of suboptimal quality [41]. Our data indicated that school-aged children who received their most recent deworming treatment from a chemist were more likely to be infected with *Trichuris spp* than those who obtained their treatment from a clinic or at school. While this may reflect the use of different (and variably effective) drug brands or drug qualities—for example, levamisole, widely available in this setting, is less effective against *Trichuris spp* than albendazole or mebendazole [44], and its use in individual but not school-based deworming may have led to these differences—it may also be reflective of the spectrum of reasons individuals take deworming medications. For example, drugs at school may have been administered as MDA, without regard to actual infection status, while drugs from chemists may have been purchased with the intention of treating a known infection. If this is indeed the case, children whose parents purchased drugs from the chemist may be at higher risk for infection. Information about how and why parents obtain deworming medications for their children outside of MDA would be useful in answering these questions.

Beyond the potential individual effects of suboptimal treatment, use of suboptimal drugs on a population level may promote the selection of worms resistant to anthelminthic treatments [41, 45]. Veterinary data demonstrate the rapid spread of benzimidazole resistance in animal populations treated with the drug (reviewed in [45]). While there are few data to suggest that anthelminthic resistance is a widespread problem in humans at present, our inability to accurately determine the true frequency of deworming makes it more difficult to monitor drug efficacy and assess the potential for anthelminthic resistance [46]. Surveys of medications available and doses recommended by chemists would provide information on the occurrence and frequency of suboptimal drug treatment, and allow for opportunities to correct common problems.

As indicated from our data, in addition to NGO- and other-entity-driven deworming, individual treatment may also be common. Although reporting of individual treatment is impractical, individual treatment may prove particularly important in the‘endgame’ of STH control, when STH infections may still be present, but at too low a prevalence to warrant MDA [18]. In such settings, continued suppression of STH transmission will likely require the availability of high-quality and low-cost deworming drugs, health-seeking behavior to access these drugs on an individual basis, and improved sanitation and hygiene. In addition, individual treatment will remain important for infected subpopulations who often are not targeted for routine STH treatment, such as adult men, and, currently, women of child-bearing age, who are at increased risk of hookworm-related anemia [47–49]. Understanding the factors that drive self-treatment can serve to prepare public health officials for the STH’endgame.’

Despite the challenges associated with unprogrammed deworming, it currently fills a need in places where programmed deworming is not occurring. Although Nairobi is not eligible for school-based MDA due to its overall low prevalence of STH infection in school-aged children, Kibera, an impoverished slum inside the city limits, clearly represents a pocket of high prevalence of STH infection, likely due to its very poor water quality and poor sanitation [50]. For school-aged children in this area, the unprogrammed deworming carried out by the local government in partnership with NGOs fills an otherwise unmet need. In addition, for preschool-aged children in Kibera, there is no programmed treatment, including MDA to treat lymphatic filariasis (although IMCI deworming guidelines are implemented in clinical settings) [37]. Unprogrammed treatment partially fills this gap, at no cost to national governments. It is important to encourage further understanding of how, how much, and why unprogrammed treatment occurs, to assist in evaluating its contribution to STH control both inside and outside the context of national STH control programs.

To this end, the STH community must develop validated, comprehensive, and flexible tools to evaluate the frequency, source, and impact of unprogrammed deworming received whenever MDA coverage or effectiveness surveys are implemented. These tools should include questions about the number of times deworming medication was received outside of the context of MDA during the past year, where the drug was sourced from (if known), the type of drug obtained, and, among persons who choose to obtain their own deworming medications, when and why they opted to do so. Sources of drugs provided by NGOs should also be investigated and a sample of locally-available deworming medications tested, to evaluate their effectiveness at delivering the promised results. Different approaches may be required in different settings to confirm the extent of unprogrammed deworming and the source of drugs for different target groups. The variety of drug sources and informants involved in providing and reporting unprogrammed deworming adds a layer of complexity to its accurate assessment. It was not possible in our study to ascertain the veracity of parent-reported deworming; it is possible that other locally-available (and perhaps locally specific) deworming medication sources exist, and setting-specific tools should be developed to assess these during formal evaluations of STH MDA programs, perhaps including components of record review from NGOs and schools as well as individual recall.

In addition to possible recall challenges, potential limitations to our analysis include drug misclassification by respondents: what respondents thought was a deworming drug may have been something else, and what they thought was a drug for a different purpose may have been an anthelminthic. Future studies should verify agreement between the answer to deworming questions without visual aids and the answer when mothers are shown the different deworming drugs and preparations available in the area and asked which one, if any, their child received. In addition, this study took place in an area where a national government-sponsored deworming program was not occurring; were such a program taking place, the frequency of unprogrammed deworming, particularly by NGOs partnering with local government, might have been lower. However, non-school-based receipt of deworming medications accounted for approximately 60% of deworming drugs in this study, suggesting that individual deworming might continue. Partially as a result of the findings in this study, there are now discussions about implementing school-based anthelminthic MDA as part of the national program in parts of Nairobi. Should this occur, a repeat of this evaluation at that time will help shed light on the frequency of unprogrammed treatment while national government-sponsored MDA is occurring. Finally, as an urban African slum, Kibera is unusual in that it serves as a setting for multiple studies and NGO-based interventions. Due to the abundance of NGOs in Kibera, children living there may be more likely to have received NGO-sponsored unprogrammed deworming compared with children living in other urban slums.

In summary, unprogrammed deworming is substantial in Kibera, Kenya. Anecdotal and limited published evidence suggests that unprogrammed deworming is both prevalent and widespread. STH control programs must develop ways to determine the extent, impact, and patterns of unprogrammed deworming to inform guidelines and rational approaches to STH control.

## Supporting Information

S1 Checklist STROBE Checklist. (DOC)

## References

[1] de Silva NR, Brooker S, Hotez PJ, Montresor A, Engels D, Savioli L. Soil-transmitted helminth infections: updating the global picture. Trends in parasitology 2003; 19(12): 547–51. 14642761 10.1016/j.pt.2003.10.002

[2] Odiere MR, Rawago FO, Ombok M, et al High prevalence of schistosomiasis in Mbita and its adjacent islands of Lake Victoria, western Kenya. Parasites & vectors 2012; 5: 278. 23206383 10.1186/1756-3305-5-278 PMC3523971

[3] Martin J, Keymer A, Isherwood RJ, Wainwright SM. The prevalence and intensity of *Ascaris lumbricoides* infections in Moslem children from northern Bangladesh. Transactions of the Royal Society of Tropical Medicine and Hygiene 1983; 77(5): 702–6. 6362124 10.1016/0035-9203(83)90210-9

[4] Elkins DB, Haswell-Elkins M, Anderson RM. The importance of host age and sex to patterns of reinfection with *Ascaris lumbricoides* following mass anthelmintic treatment in a South Indian fishing community. Parasitology 1988; 96 (Pt 1): 171–84. 3362574 10.1017/s0031182000081749

[5] Smith H, Dekaminsky R, Niwas S, Soto R, Jolly P. Prevalence and intensity of infections of *Ascaris lumbricoides* and *Trichuris trichiura* and associated socio-demographic variables in four rural Honduran communities. Memorias do Instituto Oswaldo Cruz 2001; 96(3): 303–14. 11313635 10.1590/s0074-02762001000300004

[6] Thein H, Than S, Myat Lay K. Control of ascariasis through age-targeted chemotherapy: impact of 6-monthly chemotherapeutic regimens. Bulletin of the World Health Organization 1990; 68(6): 747–53. 2150011 PMC2393170

[7] Brooker S, Peshu N, Warn PA, et al The epidemiology of hookworm infection and its contribution to anaemia among pre-school children on the Kenyan coast. Transactions of the Royal Society of Tropical Medicine and Hygiene 1999; 93(3): 240–6. 10492749 10.1016/s0035-9203(99)90007-x

[8] Layrisse M, Roche M. The Relationship between Anemia and Hookworm Infection. Results of Surveys of Rural Venezuelan Population. American journal of hygiene 1964; 79: 279–301. 14159948 10.1093/oxfordjournals.aje.a120383

[9] Stoltzfus RJ, Albonico M, Chwaya HM, et al Hemoquant determination of hookworm-related blood loss and its role in iron deficiency in African children. The American journal of tropical medicine and hygiene 1996; 55(4): 399–404. 8916795 10.4269/ajtmh.1996.55.399

[10] Mahalanabis D, Jalan KN, Maitra TK, Agarwal SK. Vitamin A absorption in ascariasis. The American journal of clinical nutrition 1976; 29(12): 1372–5. 998548 10.1093/ajcn/29.12.1372

[11] Stephenson LS, Latham MC, Kinoti SN, Kurz KM, Brigham H. Improvements in physical fitness of Kenyan schoolboys infected with hookworm, *Trichuris trichiura* and *Ascaris lumbricoides* following a single dose of albendazole. Transactions of the Royal Society of Tropical Medicine and Hygiene 1990; 84(2): 277–82. 2389321 10.1016/0035-9203(90)90286-n

[12] Callender JE, Walker SP, Grantham-McGregor SM, Cooper ES. Growth and development four years after treatment for the *Trichuris* dysentery syndrome. Acta paediatrica 1998; 87(12): 1247–9. 9894824 10.1080/080352598750030924

[13] Nokes C, Grantham-McGregor SM, Sawyer AW, Cooper ES, Robinson BA, Bundy DA. Moderate to heavy infections of *Trichuris trichiura* affect cognitive function in Jamaican school children. Parasitology 1992; 104 (Pt 3): 539–47. 1641252 10.1017/s0031182000063800

[14] Adams EJ, Stephenson LS, Latham MC, Kinoti SN. Physical activity and growth of Kenyan school children with hookworm, *Trichuris trichiura* and *Ascaris lumbricoides* infections are improved after treatment with albendazole. The Journal of nutrition 1994; 124(8): 1199–206. 8074755 10.1093/jn/124.8.1199

[15] Shang Y, Tang LH, Zhou SS, Chen YD, Yang YC, Lin SX. Stunting and soil-transmitted-helminth infections among school-age pupils in rural areas of southern China. Parasites & vectors 2010; 3: 97. 20942948 10.1186/1756-3305-3-97 PMC2965140

[16] Stephenson LS, Latham MC, Adams EJ, Kinoti SN, Pertet A. Physical fitness, growth and appetite of Kenyan school boys with hookworm, *Trichuris trichiura* and *Ascaris lumbricoides* infections are improved four months after a single dose of albendazole. The Journal of nutrition 1993; 123(6): 1036–46. 8505663 10.1093/jn/123.6.1036

[17] de Silva NR, Guyatt HL, Bundy DA. Worm burden in intestinal obstruction caused by *Ascaris lumbricoides* . Tropical medicine & international health: TM & IH 1997; 2(2): 189–90. 9472304 10.1046/j.1365-3156.1997.d01-241.x

[18] World Health Organization. Helminth control in school-age children: A guide for managers of control programmes. 2011.

[19] World Health Organization. Preventive chemotherapy in human helminthiasis. 2006.

[20] Assembly WH. WHA54.19 Schistosomiasis and soil-transmitted helminth infections. 2001. 11268509

[21] London Declaration on Neglected Tropical Diseases. 2012.

[22] Kabaka S, Wanza Kisia C. National deworming program: Kenya’s experience. 2011.

[23] Brooker S, Clements AC, Bundy DA. Global epidemiology, ecology and control of soil-transmitted helminth infections. Advances in parasitology 2006; 62: 221–61. 16647972 10.1016/S0065-308X(05)62007-6 PMC1976253

[24] Pullan RL, Gething PW, Smith JL, et al Spatial modelling of soil-transmitted helminth infections in Kenya: a disease control planning tool. PLoS neglected tropical diseases 2011; 5(2): e958 10.1371/journal.pntd.0000958 21347451 PMC3035671

[25] Brooker S, Kabatereine NB, Smith JL, et al An updated atlas of human helminth infections: the example of East Africa. International journal of health geographics 2009; 8: 42 10.1186/1476-072X-8-42 19589144 PMC2714505

[26] Mwandawiro CS, Nikolay B, Kihara JH, et al Monitoring and evaluating the impact of national school-based deworming in Kenya: study design and baseline results. Parasites & vectors 2013; 6: 198. 23829767 10.1186/1756-3305-6-198 PMC3723516

[27] Gallo K, Mikhailov A, Hailemeskal MB, Koporc K, Mbabazi PS, Addiss D. Contributions of Non-Governmental Organizations to WHO Targets for Control of Soil-Transmitted Helminthiases. The American journal of tropical medicine and hygiene 2013. 10.4269/ajtmh.13-0277 PMC3854897 24166039

[28] Anderson RM, Truscott JE, Pullan RL, Brooker SJ, Hollingsworth TD. How effective is school-based deworming for the community-wide control of soil-transmitted helminths? PLoS neglected tropical diseases 2013; 7(2): e2027 10.1371/journal.pntd.0002027 23469293 PMC3585037

[29] Brooker S, Kabatereine NB, Fleming F, Devlin N. Cost and cost-effectiveness of nationwide school-based helminth control in Uganda: intra-country variation and effects of scaling-up. Health policy and planning 2008; 23(1): 24–35. 18024966 10.1093/heapol/czm041 PMC2637386

[30] Leslie J, Garba A, Oliva EB, et al Schistosomiasis and soil-transmitted helminth control in Niger: cost effectiveness of school based and community distributed mass drug administration [corrected]. PLoS neglected tropical diseases 2011; 5(10): e1326 10.1371/journal.pntd.0001326 22022622 PMC3191121

[31] Parker M, Allen T. Does mass drug administration for the integrated treatment of neglected tropical diseases really work? Assessing evidence for the control of schistosomiasis and soil-transmitted helminths in Uganda. Health research policy and systems / BioMed Central 2011; 9: 3 10.1186/1478-4505-9-3 21211001 PMC3024987

[32] Mwanthi MA, Kinoti MK, Wamae AW, Ndonga M, Migiro PS. Prevalence of intestinal worm infections among primary school children in Nairobi City, kenyA. East African journal of public health 2008; 5(2): 86–9. 19024416

[33] Davis SM, Worrell CM, Wiegand RE, et al Soil-Transmitted Helminths in Pre-School-Aged and School-Aged Children in an Urban Slum: A Cross-Sectional Study of Prevalence. The American journal of tropical medicine and hygiene 2014; in press. 10.4269/ajtmh.14-0060 PMC4228865 25157123

[34] Hafiz I, Berhan M, Keller A, et al School-based mass distributions of mebendazole to control soil-transmitted helminthiasis in the Munshiganj and Lakshmipur districts of Bangladesh: An evaluation of the treatment monitoring process and knowledge, attitudes, and practices of the population. Acta tropica 2013. 10.1016/j.actatropica.2013.12.010 24370675

[35] Menzies SK, Rodriguez A, Chico M, et al Risk Factors for Soil-Transmitted Helminth Infections during the First 3 Years of Life in the Tropics; Findings from a Birth Cohort. PLoS neglected tropical diseases 2014; 8(2): e2718 10.1371/journal.pntd.0002718 24587469 PMC3937274

[36] Albonico M, Allen H, Chitsulo L, Engels D, Gabrielli AF, Savioli L. Controlling soil-transmitted helminthiasis in pre-school-age children through preventive chemotherapy. PLoS neglected tropical diseases 2008; 2(3): e126 10.1371/journal.pntd.0000126 18365031 PMC2274864

[37] World Health Organization. Integrated Management of Childhood Illness Chart Booklet. 2014.

[38] Belizario VY Jr., Totanes FI, de Leon WU, Ciro RN, Lumampao YF. Sentinel Surveillance of Soil-Transmitted Helminthiasis in Preschool-Aged and School-Aged Children in Selected Local Government Units in the Philippines: Follow-up Assessment. Asia-Pacific journal of public health / Asia-Pacific Academic Consortium for Public Health 2013. 10.1177/1010539513483825 23572379

[39] Mohammed K, Deb RM, Stanton MC, Molyneux DH. Soil transmitted helminths and scabies in Zanzibar, Tanzania following mass drug administration for lymphatic filariasis—a rapid assessment methodology to assess impact. Parasites & vectors 2012; 5: 299. 23259465 10.1186/1756-3305-5-299 PMC3543323

[40] Steinmann P, Utzinger J, Du ZW, et al Efficacy of single-dose and triple-dose albendazole and mebendazole against soil-transmitted helminths and Taenia spp.: a randomized controlled trial. PloS one 2011; 6(9): e25003 10.1371/journal.pone.0025003 21980373 PMC3181256

[41] Vercruysse J, Behnke JM, Albonico M, et al Assessment of the anthelmintic efficacy of albendazole in school children in seven countries where soil-transmitted helminths are endemic. PLoS neglected tropical diseases 2011; 5(3): e948 10.1371/journal.pntd.0000948 21468309 PMC3066140

[42] Supali T, Djuardi Y, Bradley M, Noordin R, Ruckert P, Fischer PU. Impact of six rounds of mass drug administration on brugian filariasis and soil-transmitted helminth infections in eastern indonesia. PLoS neglected tropical diseases 2013; 7(12): e2586 10.1371/journal.pntd.0002586 24349595 PMC3861187

[43] Strunz EC, Addis D, Stocks M, Ogden S, Utzinger J, Freeman MC. Water, sanitation, hygiene and soil-transmitted helminth infection: A systematic review and meta-analysis. PLoS Med 2014; in press. 10.1371/journal.pmed.1001620 PMC3965411 24667810

[44] Albonico M, Crompton DW, Savioli L. Control strategies for human intestinal nematode infections. Advances in parasitology 1999; 42: 277–341. 10050275 10.1016/s0065-308x(08)60151-7

[45] Geerts S, Gryseels B. Drug resistance in human helminths: current situation and lessons from livestock. Clinical microbiology reviews 2000; 13(2): 207–22. 10755998 10.1128/cmr.13.2.207-222.2000 PMC100151

[46] World Health Organization Assessing the Efficacy of Anthelminthic Drugs Against Schistosomiases and Soil-Transmitted Helminthiases. Geneva, Switzerland: WHO, 2013.

[47] Casey GJ, Montresor A, Cavalli-Sforza LT, et al Elimination of iron deficiency anemia and soil transmitted helminth infection: evidence from a fifty-four month iron-folic acid and de-worming program. PLoS neglected tropical diseases 2013; 7(4): e2146 10.1371/journal.pntd.0002146 23593517 PMC3623698

[48] Crompton DW, Nesheim MC. Nutritional impact of intestinal helminthiasis during the human life cycle. Annual review of nutrition 2002; 22: 35–59. 12055337 10.1146/annurev.nutr.22.120501.134539

[49] Dreyfuss ML, Stoltzfus RJ, Shrestha JB, et al Hookworms, malaria and vitamin A deficiency contribute to anemia and iron deficiency among pregnant women in the plains of Nepal. The Journal of nutrition 2000; 130(10): 2527–36. 11015485 10.1093/jn/130.10.2527

[50] Karanja JM, Ng’ang’a E. Sanitation and Hygiene in Kibera Slums, Nairobi. Helsinki, Finland: Helsinki Metropolia University of Applied Sciences, 2008.

