# Supplementary material for: Unprogrammed Deworming in the Kibera Slum, Nairobi: Implications for Control of Soil-Transmitted Helminthiases
Source: PLoS Negl Trop Dis. 2015 Mar 12;9(3):e0003590. doi: 10.1371/journal.pntd.0003590 (PMC4357447; doi:10.1371/journal.pntd.0003590)
Supplement: S1 Checklist — (DOC) [file pntd.0003590.s001.doc]

STROBE Statement—Checklist of items that should be included in reports of ***cross-sectional studies***

|  | Item No | Recommendation |
| --- | --- | --- |
| **Title and abstract** | 1 | (*a*) Indicate the study’s design with a commonly used term in the title or the abstract  **The abstract methods section indicates a cross-sectional study design.** |
|  |  | (*b*) Provide in the abstract an informative and balanced summary of what was done and what was found  **The abstract describes the study and rationale, the survey methods, and the more relevant results, and ends with two sentences describing the key findings.** |
| Introduction | | |
| Background/rationale | 2 | Explain the scientific background and rationale for the investigation being reported  **Background is provided in the Introduction on the WHO guidelines for deworming of school-aged children and current status in Kenya, as well as what is known about unprogrammed deworming at present.** |
| Objectives | 3 | State specific objectives, including any prespecified hypotheses  **The objective (to describe unprogrammed deworming among children in an ubran slum in Kenya) is stated in the last paragraph of the Introduction.** |
| Methods | | |
| Study design | 4 | Present key elements of study design early in the paper  **The study design is described in the first paragraph of the Methods.** |
| Setting | 5 | Describe the setting, locations, and relevant dates, including periods of recruitment, exposure, follow-up, and data collection  **The setting, dates, locations, and data collection are described in the first paragraph of the Methods.** |
| Participants | 6 | (*a*) Give the eligibility criteria, and the sources and methods of selection of participants  **The eligibility criteria and selection methods are described in the first paragraph of the Methods.** |
| Variables | 7 | Clearly define all outcomes, exposures, predictors, potential confounders, and effect modifiers. Give diagnostic criteria, if applicable  **The outcomes (triplicate Kato-Katz tests with any positive) are described in the first paragraph in the Methods, but are described in another (referenced) recent paper in detail. Questions about exposures (receiving an anthelmintic drug) are described in the same paragraph.** |
| Data sources/ measurement | 8* | For each variable of interest, give sources of data and details of methods of assessment (measurement). Describe comparability of assessment methods if there is more than one group  **There is only one group of data collected for each variable. Their methods of collection are described in the first and second paragraph in the Methods.** |
| Bias | 9 | Describe any efforts to address potential sources of bias  **We address unavoidable potential biases (specifically, recall bias) in the limitations.** |
| Study size | 10 | Explain how the study size was arrived at  **The sampling and sample size is explained in the Methods section.** |
| Quantitative variables | 11 | Explain how quantitative variables were handled in the analyses. If applicable, describe which groupings were chosen and why  **Quantitative variables such as drug coverage are described in the second paragraph of the Methods.** |
| Statistical methods | 12 | (*a*) Describe all statistical methods, including those used to control for confounding  **Statistical methods are explained in the last sentence of the Methods.** |
|  |  | (*b*) Describe any methods used to examine subgroups and interactions  **Not applicable.** |
|  |  | (*c*) Explain how missing data were addressed  **Missing data were excluded, as indicated in the second-to-last sentence of the Methods.** |
|  |  | (*d*) If applicable, describe analytical methods taking account of sampling strategy  **Not applicable.** |
|  |  | (*e*) Describe any sensitivity analyses  **Not applicable.** |
| Results | | |
| Participants | 13* | (a) Report numbers of individuals at each stage of study—eg numbers potentially eligible, examined for eligibility, confirmed eligible, included in the study, completing follow-up, and analysed  **For this paper, a subgroup of children from the parent study had data on deworming, and those were included in the analyses, as indicated in the first sentence of the results.** |
|  |  | (b) Give reasons for non-participation at each stage  **Not applicable.** |
|  |  | (c) Consider use of a flow diagram |
| Descriptive data | 14* | (a) Give characteristics of study participants (eg demographic, clinical, social) and information on exposures and potential confounders  **Children in this study are divided into infants, preschool-aged, and school-aged, as indicated in the Methods, and described in the first sentence of the Results.** |
|  |  | (b) Indicate number of participants with missing data for each variable of interest **This is indicated with each report of data in the Results.** |
| Outcome data | 15* | Report numbers of outcome events or summary measures  **Numbers of children dewormed and numbers receiving anthelmintic drugs are reported in the Results.** |
| Main results | 16 | (*a*) Give unadjusted estimates and, if applicable, confounder-adjusted estimates and their precision (eg, 95% confidence interval). Make clear which confounders were adjusted for and why they were included  **Unadjusted proportions for each variable of interest are reported throughout the Results. Adjustments are not applicable.** |
|  |  | (*b*) Report category boundaries when continuous variables were categorized  **Not applicable.** |
|  |  | (*c*) If relevant, consider translating estimates of relative risk into absolute risk for a meaningful time period  **Not applicable.** |
| Other analyses | 17 | Report other analyses done—eg analyses of subgroups and interactions, and sensitivity analyses  **Subgroup analyses are reported in the *School-based deworming of school-aged children in Kibera* and *NGO deworming of schools* sections.** |
| Discussion | | |
| Key results | 18 | Summarise key results with reference to study objectives  **Key results are summarized in the first paragraph of the Results.** |
| Limitations | 19 | Discuss limitations of the study, taking into account sources of potential bias or imprecision. Discuss both direction and magnitude of any potential bias  **Limitations are included at the end of the Discussion and include a sentence on how the data might have changed were national government-sponsored deworming taking place. Systematic error (bias) is unlikely to have been induced by other limitations to the study.** |
| Interpretation | 20 | Give a cautious overall interpretation of results considering objectives, limitations, multiplicity of analyses, results from similar studies, and other relevant evidence  **The overall results are interpreted cautiously in the first, third, fourth, and fifth paragraphs of the Discussion.** |
| Generalisability | 21 | Discuss the generalisability (external validity) of the study results  **The potential limitations to generalizability are discussed in the paragraph on limitations in the Discussion (second to last paragraph).** |
| Other information | | |
| Funding | 22 | Give the source of funding and the role of the funders for the present study and, if applicable, for the original study on which the present article is based  **The funding for the original study is listed after the acknowledgements.** |

*Give information separately for exposed and unexposed groups.

**Note:** An Explanation and Elaboration article discusses each checklist item and gives methodological background and published examples of transparent reporting. The STROBE checklist is best used in conjunction with this article (freely available on the Web sites of PLoS Medicine at http://www.plosmedicine.org/, Annals of Internal Medicine at http://www.annals.org/, and Epidemiology at http://www.epidem.com/). Information on the STROBE Initiative is available at www.strobe-statement.org.
